# Aspirin reverses inflammatory suppression of chondrogenesis by stabilizing YAP

**DOI:** 10.1111/cpr.13380

**Published:** 2022-12-10

**Authors:** Xudong Wang, Hongyi Liao, Yong Liu, Yunze Kang, Qingqiang Tu, Zhiwen Li, Yan Kang, Puyi Sheng, Ziji Zhang

**Affiliations:** ^1^ Department of Orthopedics the First Affiliated Hospital of Sun Yat‐sen University Guangzhou Guangdong China; ^2^ Guangdong Provincial Key Laboratory of Orthopedics and Traumatology the First Affiliated Hospital of Sun Yat‐sen University Guangzhou Guangdong China

## Abstract

Bone marrow mesenchymal stem cells (BMMSCs) transplantation methods are promising candidates for osteoarthritis (OA) treatment. However, inflammatory factors (such as TNF‐α) that occur at cell transplantation sites are critical factors that impair the effectiveness of the treatment. Previous studies have shown that aspirin (AS) had a regulatory role in stem cell differentiation. However, little is known about the role of AS on the chondrogenesis of BMMSCs. The purpose of this study is to explore the protective role of AS against the negative effects of TNF‐α on BMMSC chondrogenesis. In this study, we investigated the effects of AS and TNF‐α on BMMSCs chondrogenesis by performing the Alcian Blue staining, safranin O‐fast green staining, haematoxylin and eosin staining, and immunohistochemical staining, as well as real‐time RT‐PCR and western blot assays. Our results demonstrated that TNF‐α inhibited chondrogenic differentiation of BMMSCs by disrupting the balance of cartilage metabolism and promoting oxidative stress in BMMSCs, while AS treatment attenuated these effects. Furthermore, a detailed molecular mechanistic analysis indicated that Yes‐associated protein (YAP) played a critical regulatory role in this process. In addition, AS treatment mitigated the progression of cartilage degeneration in a mouse destabilization of the medial meniscus (DMM) model. AS alleviated the inhibitory effect of TNF‐α on chondrogenesis of BMMSCs by stabilizing YAP, which may provide new therapeutic strategies for OA treatment.

## Graphical abstract

Aspirin mitigates the progression of cartilage degeneration in a mouse DMM model. (A) A schematic of the in vivo experiment. After DMM surgery, mice were injected with aspirin or PBS for 12 weeks (*n* = 10 per group). (B) The body weights of mice from different treatment groups were measured after 12 weeks. (C) Representative H&E and safranin O‐fast green staining images of the left knee joint of mice after exposure to different treatments for 8 weeks (*n* = 5 per group). (D) The severity of the OA‐like phenotype 8 weeks after DMM surgery in (C) was analysed by the Osteoarthritis Research Society International (OARSI) score system. (E) Representative H&E and safranin O‐fast green staining images of the left knee joint of mice after exposure to different treatments for 12 weeks (*n* = 5 per group). (F) The severity of the OA‐like phenotype 12 weeks after DMM surgery in (E) was analysed by the Osteoarthritis Research Society International (OARSI) score system. (G) IHC staining of chondrogenic markers (COL2A1 and SOX9) and catabolic markers (MMP9 and MMP13) in the left knee joint of mice after different treatments for 8 weeks. (H) IHC staining scores of (G). (I) IHC staining of chondrogenic markers (COL2A1 and SOX9) and catabolic markers (MMP9 and MMP13) in the left knee joint of mice after different treatments for 12 weeks. (J) IHC staining scores of (I). *p < 0.05, **p < 0.01, ***p < 0.001. Scale bars: 0.5 mm (50× figures), 250 μm (100× figures), 100 μm (200× figures), 50 μm (400× figures).

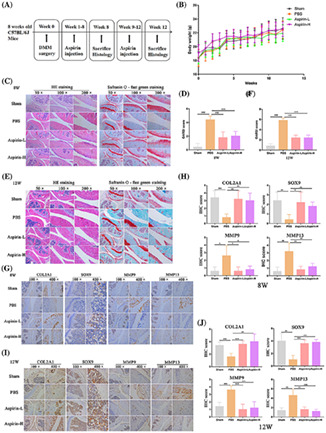

## 1 INTRODUCTION

Osteoarthritis (OA), a chronic disease characterized by degenerative changes in articular cartilage accompanied by subchondral bone sclerosis and synovitis, is the cause of both socioeconomic and personal health burdens.^1^, ^2^ With an aging global population and increasing obesity, the incidence of OA is increasing year by year.^3^ At present, OA is generally treated with conservative drugs in the early stage of the disease, and surgical treatment such as joint replacement in the later stages.^4^ However, these treatment strategies are limited by factors such as poor efficacy of drugs,^5^ large surgical trauma, and high cost. Therefore, the development of alternative therapeutic strategies is critical. In recent years, the use of bone marrow mesenchymal stem cells (BMMSCs) that differentiate into cartilage has shown multiple advantages over traditional therapies in the treatment of OA. However, although this treatment strategy has the potential for clinical application,^6^, ^7^, ^8^ its use is limited by the low differentiation rate of BMMSCs due to the presence of inflammatory factors, such as tumour necrosis factor‐alpha (TNF‐α).^9^, ^10^ Thus, the development of strategies to improve the differentiation of BMMSCs into chondrocytes in an inflammatory environment is an important issue that requires further attention.

Aspirin (AS), also known as acetylsalicylic acid, is a commonly used nonsteroidal anti‐inflammatory drug, which plays an important role in many physiological processes in the human body, such as anti‐inflammation and analgesia, and has been implicated in anti‐tumour responses and prevention of thrombosis.^11^, ^12^, ^13^, ^14^ Previous studies have shown that AS can regulate the physiological functions of mesenchymal stem cells (MSCs) in multiple ways, including inhibition of stem cell proliferation, promotion of stem cell apoptosis, promotion of myocardial differentiation and osteogenesis, and inhibition of adipogenic differentiation.^15^, ^16^, ^17^, ^18^, ^19^ However, whether AS can reverse the inhibitory effect of TNF‐α on chondrogenic differentiation of BMMSCs remains unknown. Therefore, the purpose of this study was to determine whether AS can reverse the TNF‐α‐induced chondrogenic damage of BMMSCs, in order to provide an experimental basis for the application of AS in the prevention and treatment of OA in the future.

## 2 MATERIALS AND METHODS

### 2.1 Isolation and culture of human BMMSCs

The BMMSCs used in this study were isolated and extracted from healthy volunteers as previously described.^20^, ^21^, ^22^ Isolated BMMSCs were cultured in low glycemic DMEM mediums containing 10% foetal bovine serum (FBS), 100 U/ml penicillin, and 100 mg/ml streptomycin. The culture medium was changed every 3 days.

### 2.2 Chondrogenic differentiation of BMMSCs

The high‐density hanging drop method was used to culture cartilage pellets as described previously.^20^ Pellets were cultured in the Mesenchymal Stem Cell Chondrogenic Differentiation Medium (#7551, ScienCell, USA) with or without TNF‐α (10 ng/ml) and AS (100 μM) for 7, 14, and 21 days. Cartilage pellets were collected for follow‐up experiments.

### 2.3 Antibodies and reagents

Antibodies against COL2A1, ACAN, SOX9, ADAMTS4, MMP9, MMP13, NOX1, NOX2, SOD1, SOD2, P‐YAP, and YAP were purchased from Abcam (Cambridge, UK). Antibodies against GAPDH were purchased from Cell Signaling Technology (Boston, USA). Goat anti‐rabbit IgG H&L (HRP) and Goat anti‐mouse IgG H&L (HRP) were purchased from Cell Signaling Technology (Boston, USA). TNF‐α was purchased from NovoProtein (China) and AS was purchased from MedChemExpress (New Jersey, USA).

### 2.4 Alcian Blue staining

Cartilage pellets were fixed in 4% paraformaldehyde (Phygene, Fujian, China) for 6 h, then embedded in paraffin. Paraffin‐embedded samples were sliced to a thickness of 3–5 μm and placed on glass slides. The slides were deparaffinized before samples were incubated in Alcian Blue solution (pH 2.5; Servicebio, Wuhan, China) at room temperature for 30 min. The slides were dehydrated using increasing concentrations of ethanol three times (5 min each time), cleared with xylene for 5 min, then sealed with neutral resin. Finally, samples were visualized and images captured using an Olympus BX63 microscope (Olympus, Japan).

### 2.5 Safranin O‐fast green staining

Cartilage pellets and mouse knee joint tissues were fixed in 4% paraformaldehyde for 6 h, then embedded in paraffin. Paraffin‐embedded samples were sliced to a thickness of 3–5 μm and placed on glass slides. The slides were deparaffinized before samples were incubated in Fast Green Solution (Servicebio, Wuhan, China) at room temperature for 3–5 min. Excess stain was removed by washing with water, then samples were immersed in hydrochloric acid in alcohol for 3–5 s, and incubated with Safranin O Solution (Servicebio, Wuhan, China) for 10–15 s. The slides were dehydrated using increasing concentrations of ethanol three times (5 min each time), cleared with xylene for 5 min, then sealed with neutral resin. Finally, samples were visualized and images captured using an Olympus BX63 microscope (Olympus, Japan).

### 2.6 Real‐time RT‐PCR assay

Total RNA samples were extracted from cultured BMMSCs using the Total RNA Extraction Kit (#15596018; GIBCO, USA), as per the manufacturer's instructions. Using the NanoDrop 2000 spectrophotometer (Thermo Fisher Scientific, USA) detected the RNA concentrations. The cDNAs were subsequently synthesized from 1 μg total RNA using the Evo M‐MLVRT Kit (#AG11706; Accurate Biotechnology, Hunan, China). Then, a qRT‐PCR assay was performed using the SYBR Green Pro Taq HS Premix II Kit (#AG11702; Accurate Biotechnology, Hunan, China), as per the manufacturer's instructions. The mRNA expression levels were calculated using the standard 2^−ΔΔCt^ method based on at least three biological replicates. The primer sequences of genes involved in this manuscript were shown in Table 1.

**TABLE 1:** Sequences of primers used for quantitative PCR

Gene	Primer sequence (5′‐3′)
*GAPDH*	Forward: GGAGCGAGATCCCTCCAAAAT; Reverse: GGCTGTTGTCATACTTCTCATGG
*COL2A1*	Forward: GCTGCGGATGCTCTCAATCT; Reverse: TGGACGATCAGGCGAAACC
*ACAN*	Forward: ACTCTGGGTTTTCGTGACTCT; Reverse: ACACTCAGCGAGTTGTCATGG
*SOX9*	Forward: AGCGAACGCACATCAAGAC; Reverse: CTGTAGGCGATCTGTTGGGG
*ADAMTS4*	Forward: GAGGAGGAGATCGTGTTTCCA; Reverse: CCAGCTCTAGTAGCAGCGTC
*MMP9*	Forward: TGTACCGCTATGGTTACACTCG; Reverse: GGCAGGGACAGTTGCTTCT
*MMP13*	Forward: ACTGAGAGGCTCCGAGAAATG; Reverse: GAACCCCGCATCTTGGCTT
*NOX1*	Forward: GCACACCTGTTTAACTTTGACTG; Reverse: GGACTGGATGGGATTTAGCCA
*NOX2*	Forward: AACGAATTGTACGTGGGCAGA; Reverse: GAGGGTTTCCAGCAAACTGAG
*SOD1*	Forward: GGTGGGCCAAAGGATGAAGAG; Reverse: CCACAAGCCAAACGACTTCC
*SOD2*	Forward: GCTCCGGTTTTGGGGTATCTG; Reverse: GCGTTGATGTGAGGTTCCAG
*CTGF*	Forward: CAGCATGGACGTTCGTCTG; Reverse: AACCACGGTTTGGTCCTTGG
*CYR61*	Forward: CTCGCCTTAGTCGTCACCC; Reverse: CGCCGAAGTTGCATTCCAG
*YAP*	Forward: TAGCCCTGCGTAGCCAGTTA; Reverse: TCATGCTTAGTCCACTGTCTGT

Abbreviations: ACAN, aggrecan; ADAMTS4, ADAM metallopeptidase with thrombospondin type 1 motif 4; COL2A1, collagen type II alpha 1 chain; CTGF, connective tissue growth factor; CYR61, cysteine‐rich angiogenic inducer 61; GAPDH, glyceraldehyde‐3‐phosphate dehydrogenase; MMP9, matrix metallopeptidase 9; MMP13, matrix metallopeptidase 13; NOX1, NADPH oxidase 1; NOX2, NADPH oxidase 2; SOD1, superoxide dismutase 1; SOD2, superoxide dismutase 2; SOX9, SRY‐box transcription factor 9; YAP, yes‐associated protein.

### 2.7 Western blot assay

Total protein samples were extracted from cultured BMMSCs using RIPA lysis solution (#P0013C, Beyotime, USA) containing proteinase and phosphatase inhibitors (1:100). Protein concentrations were determined using a BCA Protein Concentration Determination Kit (#P0012, Beyotime, USA), and protein samples were separated using a PAGE Gel Fast Preparation Kit (#PG112, EpiZyme, China) and transferred onto a polyvinylidene fluoride (PVDF) membrane. Membranes were blocked with 5% lipid‐free milk solution at room temperature for 60 min, then incubated with anti‐COL2A1 (1:1000), anti‐ACAN (1:1000), anti‐SOX9 (1:1000), anti‐ADAMTS4 (1:1000), anti‐MMP9 (1:1000), anti‐MMP13 (1:1000), anti‐NOX1(1:1000), anti‐NOX2 (1:1000), anti‐SOD1 (1:1000), anti‐SOD2 (1:1000), anti‐phospho‐YAP (1:1000), anti‐YAP (1:1000), and anti‐GAPDH (1:2000) antibodies overnight at 4°C. Next, membranes were incubated with secondary antibodies (1:2000) at room temperature for 60 min. Protein bands were visualized using an HRP Chemiluminescent Western Blot Detection Kit (Merck Millipore, USA).

### 2.8 Plasmids and lentivirus construction

Plasmids were obtained from Addgene (Cambridge, MA, USA), and transfection was performed using Lipofectamine 3000 reagent, according to the manufacturer's instructions. The human Yes‐associated protein (YAP) coding sequence was cloned into the lentiviral transfer plasmid pSin‐puro to construct plasmid pSin‐YAP‐FLAG. A control vector plasmid was also constructed. To silence YAP expression, the shRNA sequence was cloned into the lentiviral transfer plasmid pSin‐puro to construct pSin‐YAP‐shRNA. Control scramble‐shRNA was also constructed. Lentiviral infection was carried out as described in the previously published article.^23^

### 2.9 Hematoxylin and eosin (H&E) staining

Mouse knee joints and organ tissues (heart, liver, spleen, and kidney) of mice were fixed in 4% paraformaldehyde for 6 h and subsequently embedded in paraffin. Four‐micrometre thick sections were stained with hematoxylin (#G1005, Servicebio, Wuhan, China) for 8–10 min and with eosin (#G1005, Servicebio, Wuhan, China) for 1–2 min. Finally, samples were visualized and images captured using an Olympus BX63 microscope (Olympus, Japan).

### 2.10 Immunohistochemical (IHC) staining

Paraffin sections of the cartilage pellets and mouse knee joints were prepared for IHC experiments using the Histostain‐Plus Kit (ZSGB‐BIO, China). The antibodies included anti‐COL2A1 (1:200), anti‐SOX9 (1:500), anti‐MMP9 (1:200) and anti‐MMP13 (1:1000) antibodies. Detection was performed using a DAB Horseradish Peroxidase Color Development Kit (ZSGB‐BIO, China), and staining intensity was scored according to the previously published article.^24^

### 2.11 Animal experiments

A total of 40 specific‐pathogen‐free (SPF) female mice aged 6–8 weeks were purchased from the East Campus Animal Center of Sun Yat‐sen University (Guangzhou, China). The mice were housed in a 12‐h light/12‐h dark condition. Mice were randomly assigned to the following four groups (*n* = 10 per group): (i) Sham group, (ii) destabilization of the medial meniscus (DMM) model group (PBS group), (iii) DMM model + low dose AS group (1 mg/kg/d, 3 days per week, solvent: PBS), (iv) DMM model + high dose AS group (10 mg/kg/d, 3 days per week, solvent: PBS). An OA mice model was established using DMM surgery to the left knee joint of the mice, AS was administered by intraperitoneal injection after DMM surgery. The body weights of the mice were recorded weekly for 12 weeks. After 8 or 12 weeks of AS treatment, the left knee joint and organ tissues of the mice were removed, and H&E, Safranin O‐fast green, and IHC staining were performed on the samples. This study was approved by the Experimental Animal Ethics Committee of Sun Yat‐sen University (Animal approval certificate information ID: SYSU‐IACUC‐2021‐000788).

### 2.12 Statistical analysis

All quantitative data have been analysed using SPSS 23.0 software. The differences between the 2 and >2 groups were determined by the Student's *t*‐test and analysis of variance methods, respectively. *p* < 0.05 is statistically significant. Important notations are as follows: “*” represents *p* < 0.05, “**” represents *p* < 0.01, “***” represents *p* < 0.001.

## 3 RESULTS

### 3.1 AS alleviates TNF‐α‐induced inhibition of BMMSC cartilage pellet growth

**FIGURE 1:**
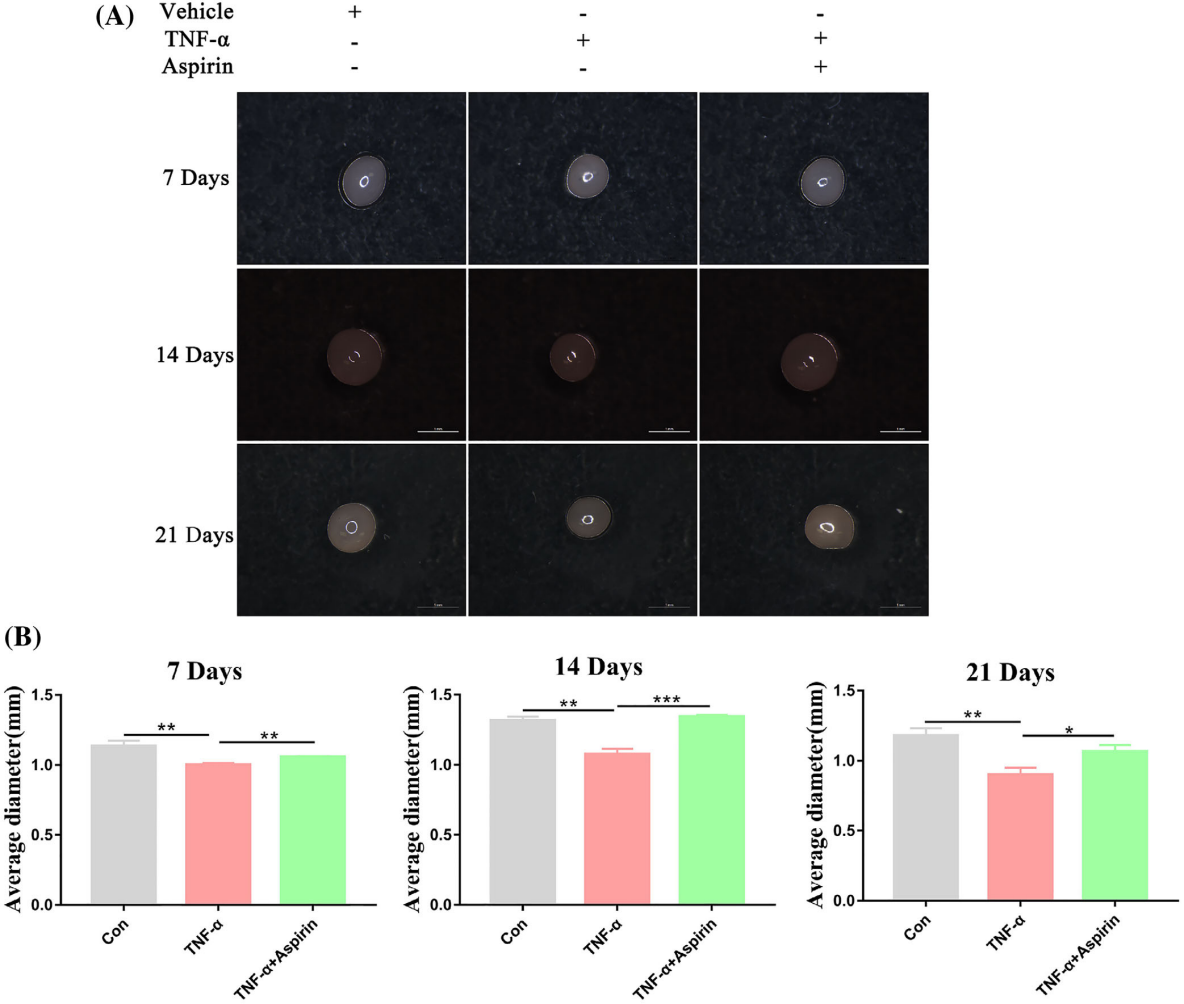
Impact of TNF‐α and aspirin on cartilage pellet development during chondrogenesis of BMMSCs. (A) Representative microscopic images showing cartilage pellets cultured under different treatment conditions for 7, 14, and 21 days. (B) Quantitative data showing the mean diameter of cartilage pellets of different treatment groups. Data in (B) are given as the mean ± SD of three independent experiments. *p < 0.05, **p < 0.01, ***p < 0.001. Scale bars: 1 mm

After 7, 14, and 21 days of BMMSC chondrogenic differentiation, we used a stereomicroscope to observe the cartilage pellets cultured from BMMSCs under different treatment conditions. We found that TNF‐α inhibited the growth of cartilage pellets on days 7, 14, and 21, whereas AS treatment alleviated this inhibitory effect (Figure 1A). The quantitative data showing the average diameter of the cartilage pellets for each treatment group are shown in Figure 1B. In conclusion, AS alleviated the inhibitory effect of TNF‐α on the differentiation of cartilage pellets from BMMSCs.

### 3.2 AS reduces the inhibitory effect of TNF‐α on the synthesis and accumulation of cartilage matrix during BMMSC chondrogenic differentiation

**FIGURE 2:**
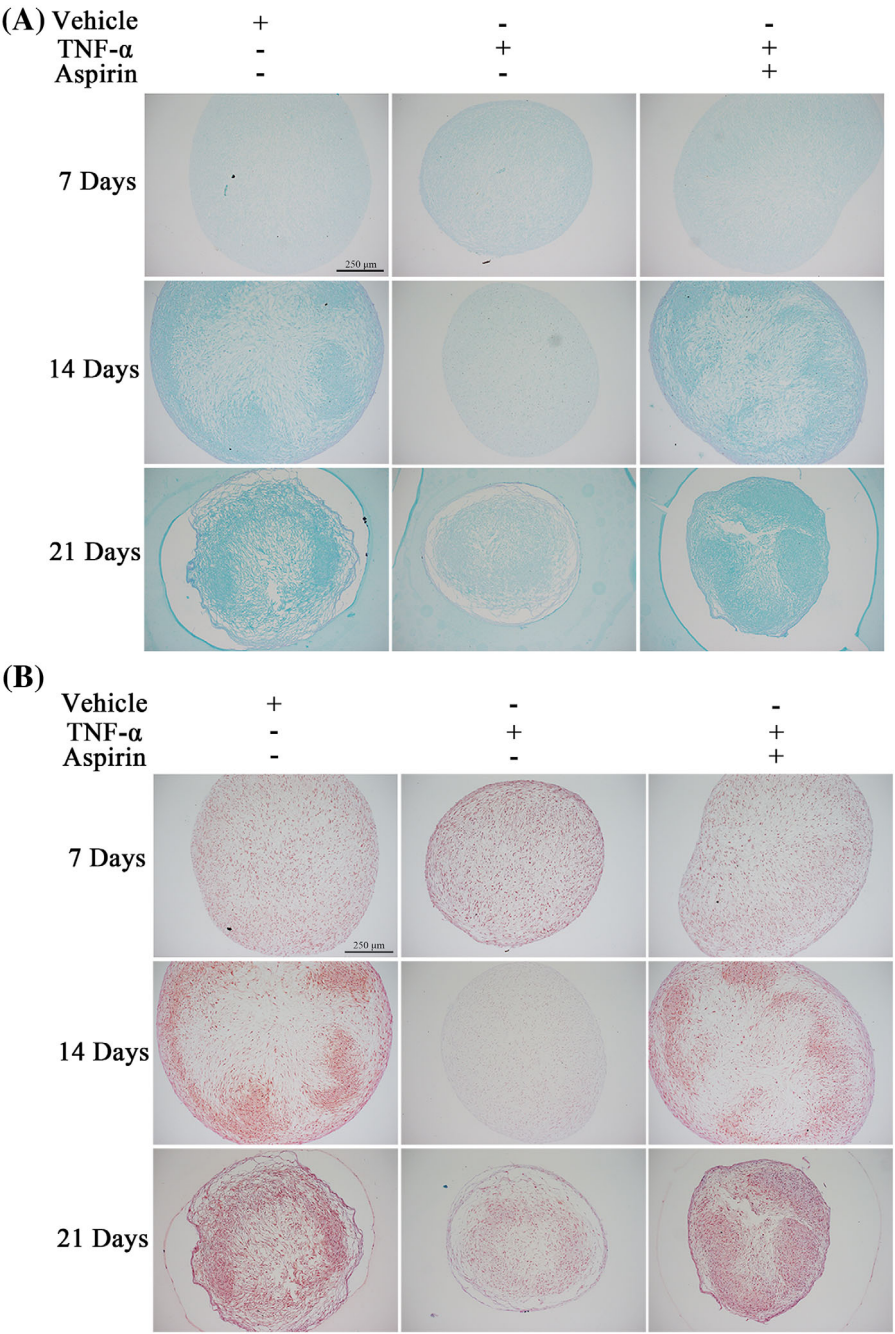
Impact of TNF‐α and aspirin on matrix accumulation during chondrogenesis of BMMSCs. (A) Representative microscopic images showing Alcian Blue staining of cartilage pellets cultured under different treatment conditions for 7, 14, and 21 days. (B) Representative microscopic images showing safranin O‐fast green staining of cartilage pellets cultured under different treatment conditions for 7, 14, and 21 days. Scale bars: 250 μm

Next, we used Alcian Blue and safranin O‐fast green staining to detect the cartilage matrix synthesis and accumulation during BMMSCs chondrogenic differentiation in the different treatment groups. We found that TNF‐α and AS treatment had no significant effect on cartilage matrix synthesis after 7 days of chondrogenic differentiation (Figure 2A,B). However, treatment with TNF‐α significantly reduced cartilage matrix synthesis on days 14 and 21, while AS treatment reversed this inhibitory effect (Figure 2A,B). These findings suggested that AS reduced the inhibitory effect of TNF‐α on cartilage matrix synthesis and accumulation during BMMSC chondrogenic differentiation.

### 3.3 AS reduces the inhibitory effect of TNF‐α on the expression of BMMSC chondrogenic differentiation markers

**FIGURE 3:**
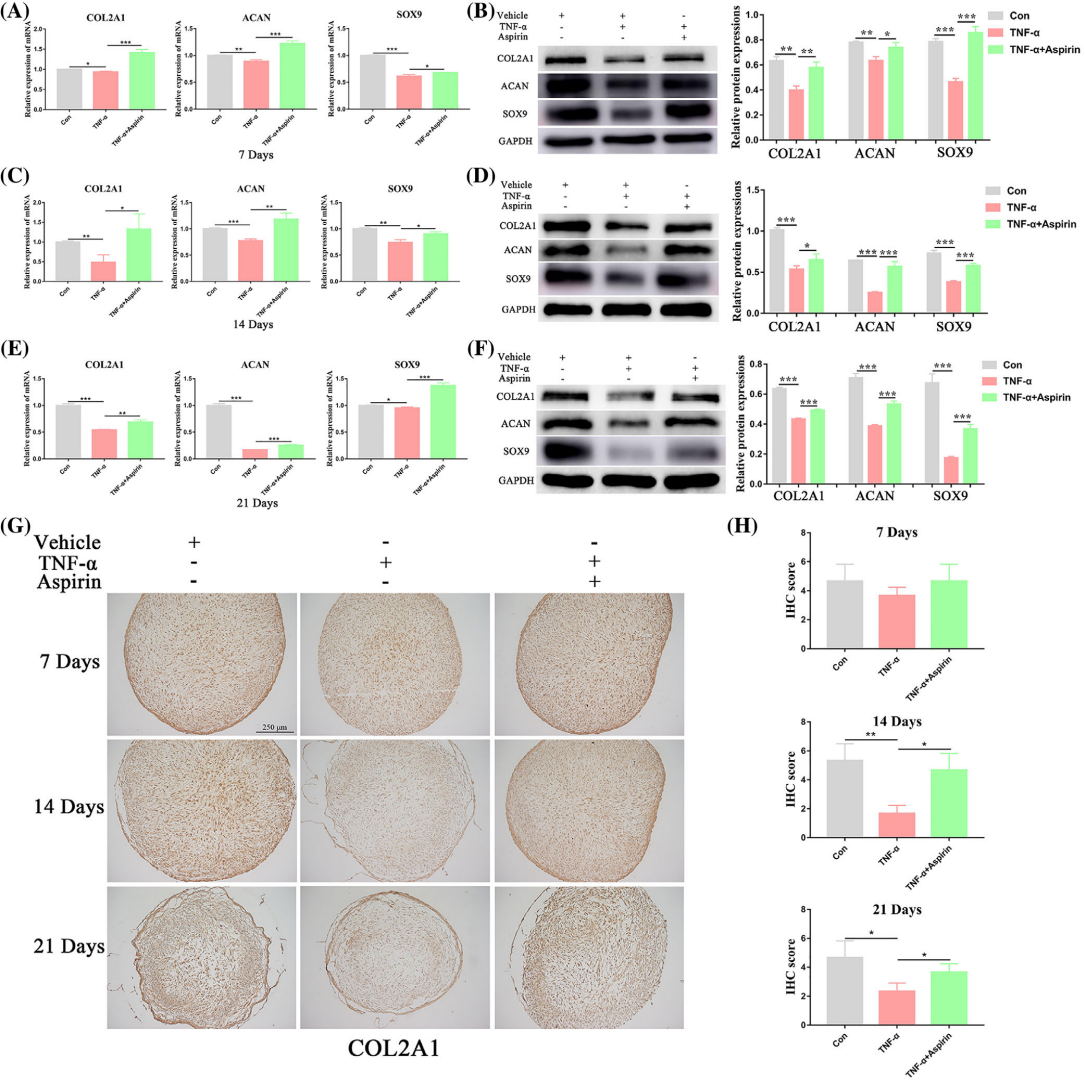
Aspirin reverses TNF‐α‐inhibited chondrogenic markers expression during chondrogenesis of BMMSCs. (A, B) Relative mRNA and protein expression levels of chondrogenic markers (COL2A1, ACAN, and SOX9) were measured in BMMSCs treated with or without TNF‐α and aspirin for 7 days by qRT‐PCR and western blot analyses, respectively. (C, D) Relative mRNA and protein expression levels of chondrogenic markers (COL2A1, ACAN, and SOX9) were measured in BMMSCs treated with or without TNF‐α and aspirin for 14 days by qRT‐PCR and western blot analyses, respectively. (E, F) Relative mRNA and protein expression levels of chondrogenic markers (COL2A1, ACAN, and SOX9) were measured in BMMSCs treated with or without TNF‐α and aspirin for 21 days by qRT‐PCR and western blot analyses, respectively. (G) IHC staining of COL2A1 in cartilage pellets cultured under different treatment conditions for 7, 14, and 21 days. (H) Quantification of the COL2A1 IHC staining data. Data in (A), (C), (E), and (H) are given as the mean ± SD of three independent experiments. ACAN, aggrecan; COL2A1, collagen type II alpha 1 chain; GAPDH, glyceraldehyde‐3‐phosphate dehydrogenase; SOX9, SRY‐box transcription factor 9. *p < 0.05, **p < 0.01, ***p < 0.001. Scale bars: 250 μm

We used RT‐PCR and western blot assays to detect the expression of chondrogenic differentiation markers (COL2A1, ACAN, SOX9) in the cartilage pellets of the different treatment groups. We found that TNF‐α significantly reduced mRNA and protein expression of these markers on 7, 14, and 21 days, while AS treatment reversed these inhibitory effects of TNF‐α (Figure 3A–F). Furthermore, immunohistochemistry (IHC) staining of COL2A1 in the different treatment groups was consistent with these findings (Figure 3G,H). These results indicated that AS reduced the inhibitory effect of TNF‐α on the expression of BMMSC chondrogenic differentiation markers.

### 3.4 AS alleviates the stimulatory effect of TNF‐α on the expression of catabolic markers of BMMSC chondrogenic differentiation

**FIGURE 4:**
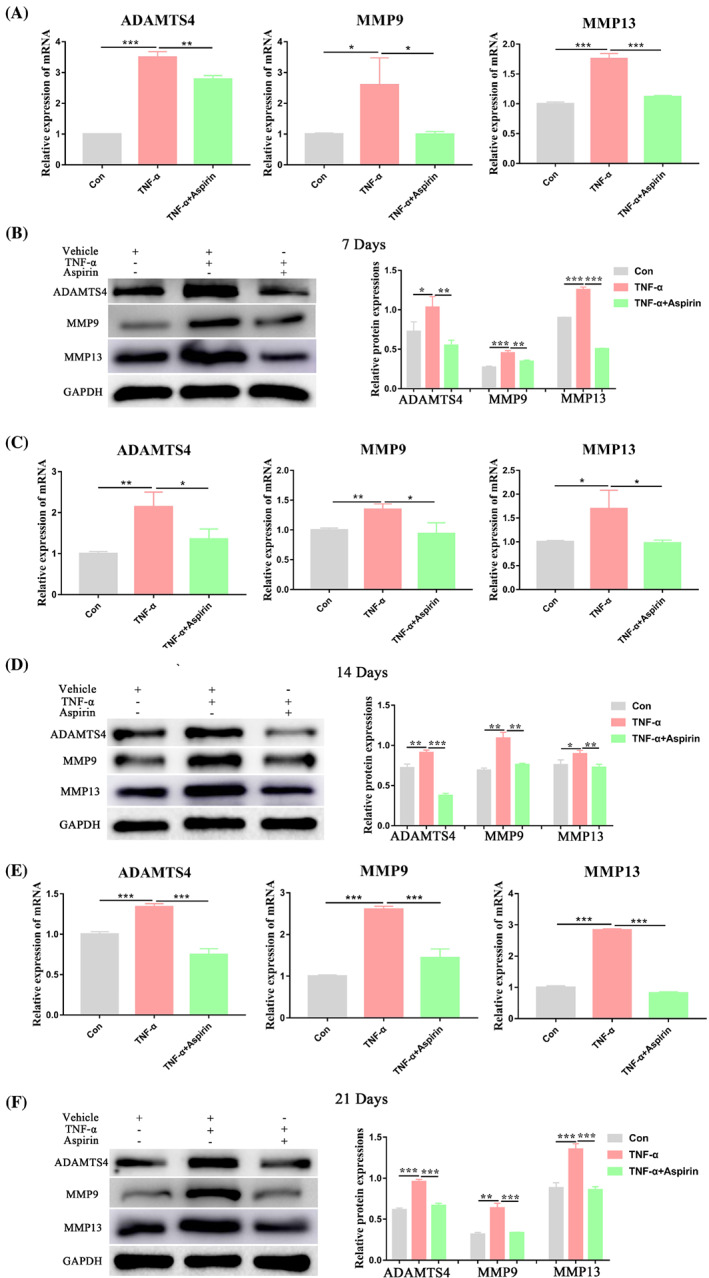
Aspirin reversed TNF‐α‐promoted catabolic markers expression during chondrogenesis of BMMSCs. (A, B) Relative mRNA and protein expression levels of catabolic markers (ADAMTS4, MMP9, and MMP13) were measured in BMMSCs treated with or without TNF‐α and aspirin for 7 days by qRT‐PCR and western blot analyses, respectively. (C, D) Relative mRNA and protein expression levels of catabolic markers (ADAMTS4, MMP9, and MMP13) were measured in BMMSCs treated with or without TNF‐α and aspirin for 14 days by qRT‐PCR and western blot analyses, respectively. (E, F) Relative mRNA and protein expression levels of catabolic markers (ADAMTS4, MMP9, and MMP13) were measured in BMMSCs treated with or without TNF‐α and aspirin for 21 days by qRT‐PCR and western blot analyses, respectively. Data in A, C, and E are given as the mean ± SD of three independent experiments. ADAMTS4, ADAM metallopeptidase with thrombospondin type 1 motif 4; GAPDH, glyceraldehyde‐3‐phosphate dehydrogenase; MMP9, matrix metallopeptidase 9; MMP13, matrix metallopeptidase 13. *p < 0.05, **p < 0.01, ***p < 0.001

In this part of the study, we detected mRNA and protein expression of catabolic markers (ADAMTS4, MMP9, MMP13) in the cartilage pellets of different treatment groups. We found that TNF‐α significantly promoted the mRNA and protein expression of these markers on 7, 14, and 21 days. While treatment with AS reduced this stimulatory effect (Figure 4A–F), demonstrating that AS reduced the stimulatory effects of TNF‐α on the expression of catabolic markers of BMMSC chondrogenic differentiation.

### 3.5 AS alleviates the stimulatory effects of TNF‐α on oxidative stress levels during chondrogenic differentiation of BMMSCs

**FIGURE 5:**
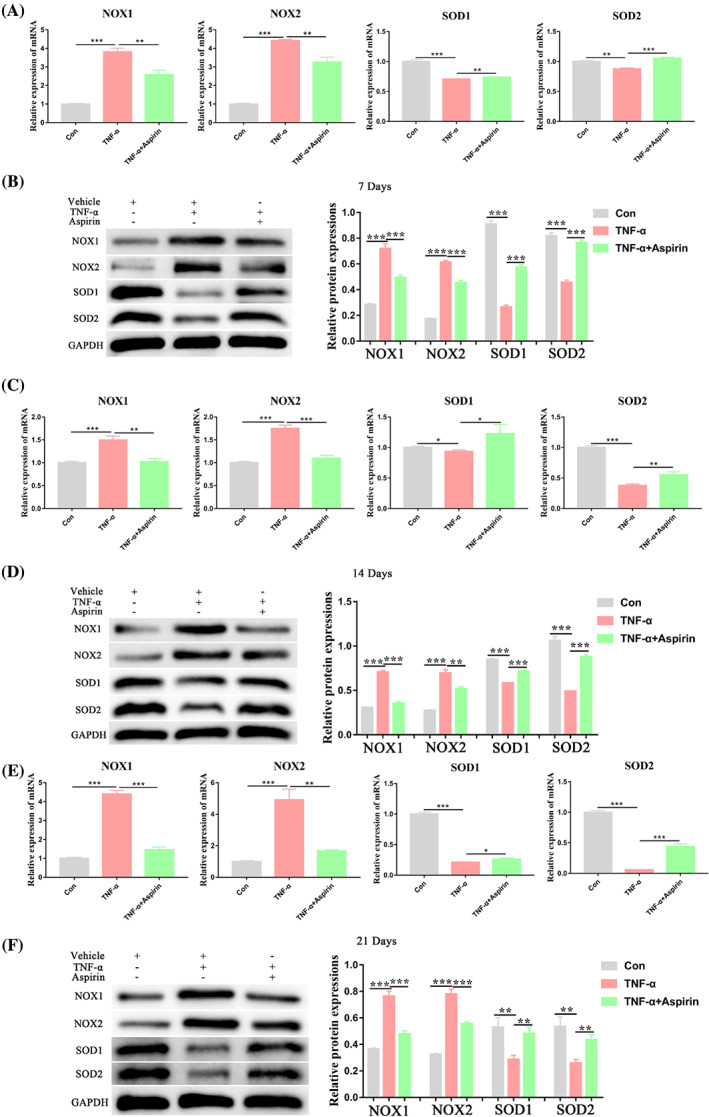
Impact of TNF‐α and aspirin on oxidative stress markers expression during chondrogenesis of BMMSCs. (A, B) Relative mRNA and protein expression levels of oxidative stress markers (NOX1, NOX2, SOD1, and SOD2) were measured in BMMSCs treated with or without TNF‐α and aspirin for 7 days by qRT‐PCR and western blot analyses, respectively. (C, D) Relative mRNA and protein expression levels of oxidative stress markers (NOX1, NOX2, SOD1, and SOD2) were measured in BMMSCs treated with or without TNF‐α and aspirin for 14 days by qRT‐PCR and western blot analyses, respectively. (E, F) Relative mRNA and protein expression levels of oxidative stress markers (NOX1, NOX2, SOD1, and SOD2) were measured in BMMSCs treated with or without TNF‐α and aspirin for 21 days by qRT‐PCR and western blot analyses, respectively. Data in (A), (C), and (E) are given as the mean ± SD of three independent experiments. GAPDH, glyceraldehyde‐3‐phosphate dehydrogenase; NOX1, NADPH oxidase 1; NOX2, NADPH oxidase 2; SOD1, superoxide dismutase 1; SOD2, superoxide dismutase 2. *p < 0.05, **p < 0.01, ***p < 0.001

Because TNF‐α is an inflammatory factor that may affect oxidative stress levels in stem cells, we next detected the mRNA and protein expression levels of oxidative stress markers (NOX1, NOX2, SOD1, and SOD2) in the different treatment groups. We found that TNF‐α significantly increased the expression of the oxidase markers (NOX1, NOX2), while inhibiting the expression of the antioxidant enzyme markers (SOD1, SOD2) on 7, 14, and 21 days. Treatment with AS reversed these effects (Figure 5A–F). Together, these data showed that AS alleviated the stimulatory effects of TNF‐α on oxidative stress levels during chondrogenic differentiation of BMMSCs.

### 3.6 AS reduces the inhibitory effect of TNF‐α on the chondrogenic differentiation of BMMSCs by stabilizing YAP

**FIGURE 6:**
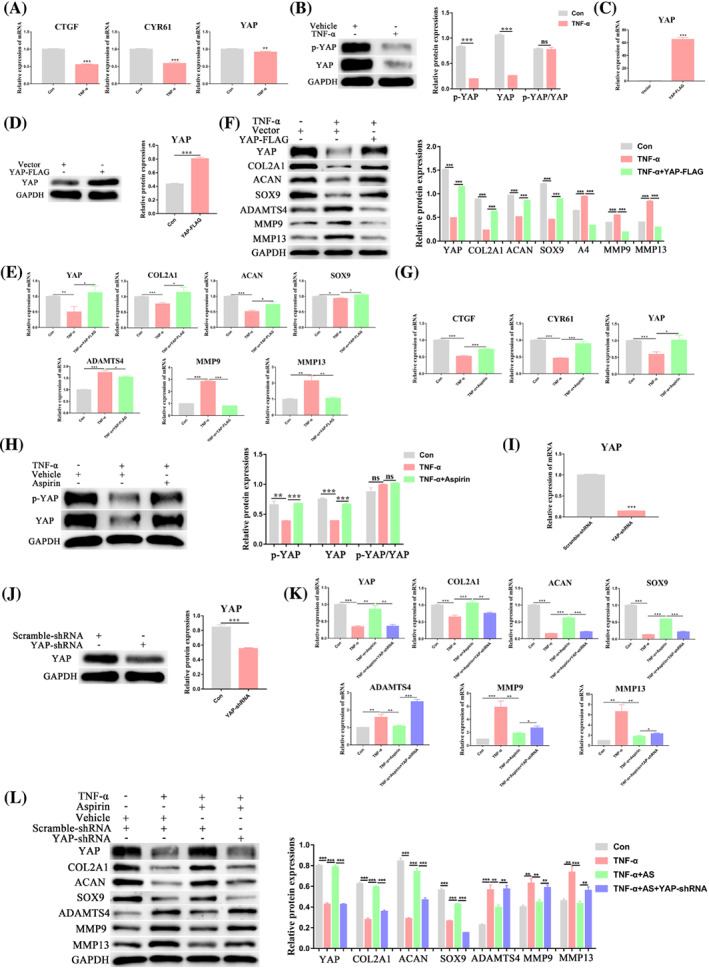
TNF‐α suppresses chondrogenesis of BMMSCs by downregulating YAP, while aspirin reverses these TNF‐α‐induced effects through stabilization of YAP. (A) The mRNA levels of YAP and key Hippo pathway target genes, including *CTGF* and *CYR61*, were measured by qPCR. (B) The p‐YAP and YAP protein expression levels were examined using western blot assays. (C, D) Stable overexpression of YAP‐FLAG or vector in BMMSCs was conducted. qPCR (C) and western blot (D) assays were performed to ensure overexpression efficiency. Chondrogenesis was then induced in BMMSCs for 14 days with or without TNFα, and expressions of YAP, COL2A1, ACAN, SOX9, ADAMTS4, MMP9, and MMP13 were detected by qPCR (E) and western blot (F). (G, H) Expressions of CTGF, CYR61 and YAP in different groups were examined by qPCR (G). The p‐YAP and YAP protein expression levels were assessed by western blot (H). (I, J) YAP expression was silenced in BMMSCs by transduction with lentivirus encoding YAP‐shRNA. Scramble‐shRNA was used as the control. qPCR (I) and western blot (J) were conducted to determine the silencing efficiency. Then, chondrogenesis differentiation was induced in BMMSCs for 14 days with or without vehicle, 10 ng/ml TNFα, and 100 μmol/L aspirin, and the expressions of YAP, COL2A1, ACAN, SOX9, ADAMTS4, MMP9, and MMP13 were detected using qPCR (K) and western blot (L) analyses. Values in A, C, E, G, I, and K are the mean ± SD of three independent experiments. ACAN, aggrecan; ADAMTS4, ADAM metallopeptidase with thrombospondin type 1 motif 4; COL2A1, collagen type II alpha 1 chain; CTGF, connective tissue growth factor; CYR61, cysteine‐rich angiogenic inducer 61; GAPDH, glyceraldehyde‐3‐phosphate dehydrogenase; MMP9, matrix metallopeptidase 9; MMP13, matrix metallopeptidase 13; SOX9, SRY‐box transcription factor 9; YAP, yes‐associated protein. *p < 0.05, **p < 0.01, ***p < 0.001

Because the Hippo pathway plays an important regulatory role in the physiological processes of stem cells, we speculated that YAP, a key component of the Hippo pathway, might be involved in mediating the inhibitory effects of TNF‐α during chondrogenic differentiation of BMMSCs. To test our hypothesis, we first detected mRNA expression of *YAP* and the typical YAP‐targeted genes *connective tissue growth factor* (*CTGF*) and *cysteine‐rich angiogenic inducer 61* (*CYR61*) after TNF‐α treatment. We found that TNF‐α reduced mRNA expression of *YAP*, *CTGF*, and *CYR61* (Figure 6A). Next, we examined p‐YAP and YAP protein expression and found that treatment with TNF‐α did not affect the p‐YAP/YAP ratio (Figure 6B).

Through YAP overexpression studies (Figure 6C,D), we found that overexpression of YAP reversed the inhibitory effect of TNF‐α on the chondrogenic synthesis markers (COL2A1, ACAN, SOX9), the antioxidant enzyme markers (SOD1, SOD2) and its stimulatory effect on the chondrogenic catabolic markers (ADAMTS4, MMP9, MMP13) and the oxidase markers (NOX1, NOX2) (Figure 6E,F, Figure S1A). These findings suggested that the inhibitory effect of TNF‐α on the chondrogenic differentiation of BMMSCs was mediated through YAP.

Subsequently, we found that AS treatment reversed the inhibitory effects of TNF‐α on *YAP*, *CTGF*, and *CYR61* mRNA expression (Figure 6G), but did not affect the p‐YAP/YAP ratio (Figure 6H). Using RNA interference to inhibit YAP expression (Figure 6I,J), we found that inhibition of YAP reversed the stimulatory effects of AS on the expression of chondrogenic differentiation synthesis markers (COL2A1, ACAN, SOX9), the antioxidant enzyme markers (SOD1, SOD2), as well as its inhibitory effects on the expression of catabolic markers (ADAMTS4, MMP9, MMP13) and the oxidase markers (NOX1, NOX2) (Figure 6K,L, Figure S1B). These results suggested that the protective effects of AS on BMMSC chondrogenic differentiation were mediated by YAP.

Taken together, our findings demonstrated that AS alleviated the inhibitory effect of TNF‐α on the chondrogenic differentiation of BMMSCs by stabilizing YAP expression.

### 3.7 AS mitigates the progression of cartilage degeneration in a DMM mouse model

**FIGURE 7:**
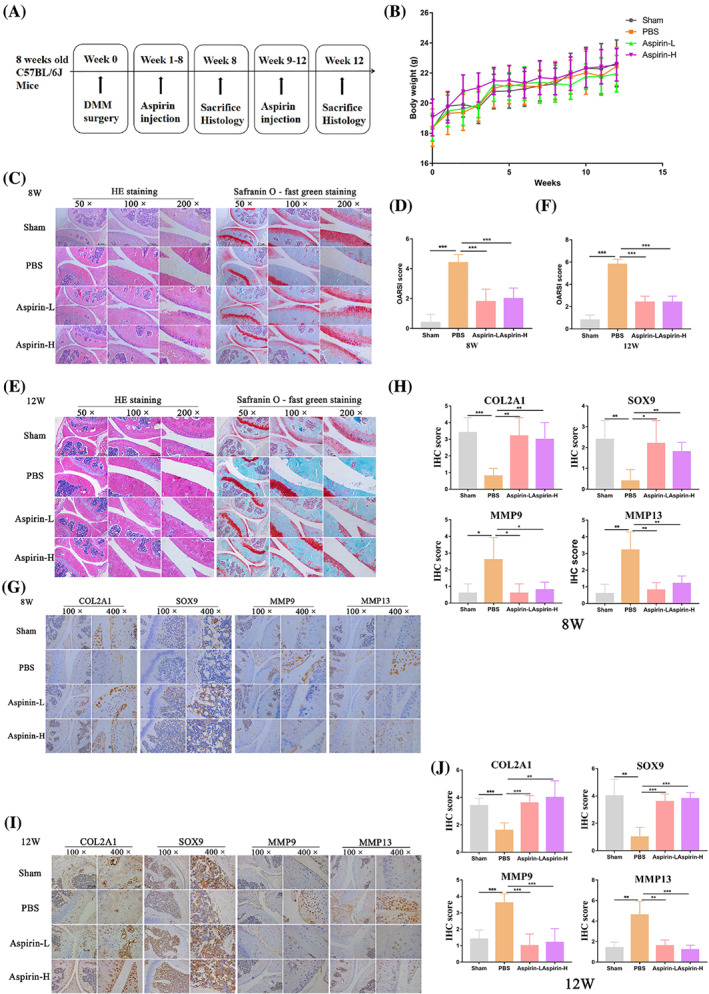
Aspirin mitigates the progression of cartilage degeneration in a mouse DMM model. A schematic of the in vivo experiment. After DMM surgery, mice were injected with aspirin or PBS for 12 weeks (*n* = 10 per group). (B) The body weights of mice from different treatment groups were measured after 12 weeks. (C) Representative H&E and safranin O‐fast green staining images of the left knee joint of mice after exposure to different treatments for 8 weeks (*n* = 5 per group). (D) The severity of the OA‐like phenotype 8 weeks after DMM surgery in (C) was analysed by the Osteoarthritis Research Society International (OARSI) score system. (E) Representative H&E and safranin O‐fast green staining images of the left knee joint of mice after exposure to different treatments for 12 weeks (*n* = 5 per group). (F) The severity of the OA‐like phenotype 12 weeks after DMM surgery in (E) was analysed by the Osteoarthritis Research Society International (OARSI) score system. (G) IHC staining of chondrogenic markers (COL2A1 and SOX9) and catabolic markers (MMP9 and MMP13) in the left knee joint of mice after different treatments for 8 weeks. (H) IHC staining scores of (G). (I) IHC staining of chondrogenic markers (COL2A1 and SOX9) and catabolic markers (MMP9 and MMP13) in the left knee joint of mice after different treatments for 12 weeks. (J) IHC staining scores of (I). COL2A1, collagen type II alpha 1 chain; MMP9, matrix metallopeptidase 9; MMP13, matrix metallopeptidase 13; SOX9, SRY‐box transcription factor 9. *p < 0.05, **p < 0.01, ***p < 0.001. Scale bars: 0.5 mm (50× figures), 250 μm (100× figures), 100 μm (200× figures), 50 μm (400× figures)

Next, we constructed an in vivo OA model by performing DMM surgery on the left knee of mice. Low (1 mg/kg/d, 3 days per week) and high (10 mg/kg/d, 3 days per week) doses of AS were administered intraperitoneally for 8 or 12 weeks post‐surgery to determine the effect of AS treatment on the progression of cartilage degeneration (Figure 7A). We found that treatment with both high and low concentrations of AS did not affect mouse body weight compared to the Sham and PBS groups (Figure 7B). Next, we performed H&E staining on heart, liver, spleen, and kidney samples collected 8 and 12 weeks after AS treatment and found that AS treatment was not toxic to these organs (Figure S2).

Eight and 12 weeks after surgical treatment of DMM, the mouse knee joints showed signs of OA, including cartilage wear and articular surface irregularities. Treatment with AS delayed the progression of knee joint cartilage degeneration in the DMM mouse model as measured by H&E staining and safranin O‐fast green staining (Figure 7C–F). IHC staining of cartilage synthesis markers (COL2A1, SOX9) and catabolic markers (MMP9, MMP13) in the knee joint tissues revealed that AS treatment led to the promotion of the expression of cartilage synthesis markers and suppression of the expression of catabolic markers in the DMM mouse model (Figure 7G,I). The quantitative data for the IHC staining of each treatment group are shown in Figure 7H,J. In conclusion, AS mitigated the progression of cartilage degeneration in the DMM mouse model by promoting cartilage synthesis and inhibiting cartilage catabolism.

## 4 DISCUSSION

The present study explored the effects of AS and TNF‐α on the chondrogenic differentiation of BMMSCs. We found that TNF‐α inhibited BMMSC chondrogenic differentiation by disrupting the balance of cartilage metabolism and promoting oxidative stress in BMMSCs, while AS treatment attenuated these effects. Mechanistically, YAP played an important regulatory role in this process. We further demonstrated in our in vivo DMM model of OA that AS treatment mitigated the progression of cartilage degeneration. Taken together, our data showed that AS alleviated the inhibitory effect of TNF‐α on BMMSC chondrogenic differentiation by stabilizing the expression of YAP. To the best of our knowledge, this is the first study to investigate the effects of AS on the chondrogenic differentiation of BMMSCs. Our study aimed to provide an experimental basis for the application of AS in the prevention and treatment of OA in the future.

The inflammatory factor TNF‐α has a negative impact on the effectiveness of stem cell therapy. Here, we found that TNF‐α inhibited chondrogenic differentiation of BMMSCs by disrupting the balance of cartilage metabolism, promoting the oxidative stress level of BMMSCs, and decreasing YAP expression. These findings are consistent with previous studies, which reported that TNF‐α can impair the physiological processes in stem cells through multiple pathways. For example, Wang et al. showed that TNF‐α impaired the stemness maintenance of BMMSCs by inhibiting the expression of YAP,^9^ while Ding et al. found that TNF‐α inhibited chondrogenic differentiation of BMMSCs, thereby promoting OA progression.^10^ TNF‐α has also been found to inhibit osteogenic differentiation of BMMSCs by promoting the production of reactive oxygen species (ROS) or degradation of SMAD1 protein.^21^, ^23^ Taken together, these studies highlighted the importance of developing therapeutic strategies to reverse the damaging effects of TNF‐α on stem cells.

AS plays an important role in regulating stem cell function. In the current study, we found that AS blocked the inhibitory effect of TNF‐α on chondrogenic differentiation of BMMSCs by stabilizing the expression of YAP, promoting cartilage metabolic homeostasis, and suppressing oxidative stress in BMMSCs. Previous studies have also demonstrated that AS can affect not only the proliferative activity of BMMSCs but also their ability to differentiate into multiple cell types. For example, Wen et al. showed that different concentrations of AS inhibited the growth of BMMSCs, as well as enhanced cardiomyocyte differentiation.^25^ In addition, AS was found to inhibit osteogenic differentiation and promote adipogenic differentiation of BMMSCs.^26^, ^27^ AS was also shown to promote cranial regeneration, as well as reduce bone loss in ovariectomized rats, and could therefore have clinical potential in the treatment of osteoporosis.^28^, ^29^, ^30^ Finally, AS was found to improve the immunomodulatory properties of BMMSCs through the 15d‐PGJ2/PPARγ/TGF‐β1 signalling axis.^31^ These studies indicate that AS has the potential to improve the efficacy of stem cell therapy.

AS also plays a critical role in OA prevention and treatment. Our study showed that AS delayed the progression of cartilage degeneration in a DMM mouse model by promoting cartilage synthesis and inhibiting cartilage catabolism. Our findings are consistent with data from a cohort study, which showed that the use of low‐dose AS was associated with reduced medial tibial cartilage loss over 2 years in patients with knee OA, suggesting that low‐dose AS may be used to slow the progression of knee OA.^16^ Meesawatsom et al. demonstrated that AS‐triggered resolvin D1 had an inhibitory effect on spinal cord damage in a rat model of chronic OA pain, and may therefore be beneficial in the treatment of inflammatory pain.^18^ AS may also have clinical potential in the treatment of secondary nociceptive hypersensitivity in OA.^19^ These studies suggested that AS may be used for OA prevention and treatment in the future.

YAP, a key molecule of the Hippo pathway, plays an important regulatory role in the physiological processes of stem cells. Here, we found that TNF‐α decreased YAP expression resulting in inhibition of BMMSCs chondrogenic differentiation, while AS treatment upregulated YAP expression, thereby delaying the inhibitory effects of TNF‐α. These findings suggest that YAP has a critical regulatory role in the chondrogenic differentiation of BMMSCs. Similarly, Wang et al. found that YAP played a regulatory role in the process of melatonin maintaining the stemness of BMMSCs^25^; Wang et al. demonstrated that BMMSC‐derived extracellular vesicles induced cartilage reconstruction in temporomandibular joint OA through the autotaxin‐YAP signalling axis,^6^ while Mao et al. found that BMMSC‐derived exosomes regulated the GPRC5A‐YAP signalling axis to improve sulfur mustard‐induced acute lung injury.^32^ Liu et al. found that the AMOT130/YAP signalling axis is an important pathway mediating micro/nano‐topography (MNT) to promote osteogenic differentiation of BMMSCs.^33^ In addition, YAP has also been shown to play an important regulatory role in reducing apoptosis and regulating osteogenic and adipogenic differentiation of BMMSCs.^34^, ^35^ These studies, together with our findings, suggested that YAP played an important regulatory role in the physiological function of stem cells.

YAP plays a significant regulatory role in stem cell chondrogenic differentiation. Yamashita et al.^36^ found that YAP knockdown could promote the chondrogenic differentiation of human‐induced pluripotent stem cells; Li et al.^37^ discovered that microtubule stabilization could inhibit YAP expression and promote the chondrogenic differentiation of synovial MSCs. Furthermore, Nie et al.^38^ found that dasatinib promoted the chondrogenic differentiation of mesenchymal stem cells by increasing the phosphorylation level of YAP. In addition to regulating the chondrogenic differentiation of stem cells, YAP has been shown to play a key role in the chondrogenesis of C3H10T1/2 and ATDC5 cells.^39^, ^40^, ^41^ Taken together, YAP plays an essential regulatory role in the process of chondrogenesis in various cell types.

YAP is also important in the regulation of oxidative stress. In this study, we discovered that YAP overexpression could partially reverse the oxidative stress‐promoting effect of TNF‐α. Silencing YAP, on the other hand, could partially reverse the down‐regulatory effect of AS on oxidative stress, indicating that YAP plays a negative regulatory role in the oxidative stress process. This is consistent with the findings of the majority of previously published literature. White et al.^42^ discovered that YAP maintains redox balance and prevents oxidative stress‐induced cell death by reducing mitochondrial respiration capacity, whereas excessive consumption of YAP can increase mitochondrial respiration and ROS accumulation, leading to oxidative stress‐induced cell death. Melatonin, according to Sun et al.,^43^ can alleviate doxorubicin‐induced mitochondrial oxidative stress injury and ferroptosis in cardiomyocytes by up‐regulating YAP expression. Cucci et al.^44^ found that by inhibiting YAP expression, Ailanthone increased oxidative stress in anti‐CDDP ovarian and bladder cancer cells. Furthermore, Huang et al.^45^ discovered that FoxO4 might exacerbate the apoptosis and oxidative stress of H9C2 cells induced by hypoxia/reoxygenation by blocking the Hippo/YAP pathway.

However, some studies have demonstrated that YAP can promote oxidative stress. Yu et al.^46^ discovered that overexpression of (Pro)renin receptor can aggravate oxidative stress and myocardial fibrosis in diabetic cardiomyopathy by increasing the expression of YAP and that YAP blockade can reverse these pathological changes. Moreover, Bai et al.^47^ established a mouse model of unilateral ureteral obstruction (UUO) and discovered that ruxolitinib treatment attenuated UUO‐induced inflammation, oxidative stress, and apoptosis by inhibiting the activation of the Akt/mTOR/Yap pathway. These studies, together with our findings, indicated that YAP played a crucial regulatory role in oxidative stress.

In this study, we chose 1 mg/kg/d and 10 mg/kg/d as the doses of AS for treatment, based on the usual doses of AS utilized in prior studies.^48^, ^49^, ^50^, ^51^ According to the findings of this study, both doses of AS alleviated cartilage degeneration in the DMM mouse model, indicating that AS has an anti‐OA effect in the DMM mouse model. According to the calculation methods in Suman et al.,^52^ 1 mg/kg/d and 10 mg/kg/d administered to mice in our study are equivalent to 22.2 mg/d and 222 mg/d in humans, which are within the range of medication doses and are safe to administer to patients in clinical practice. Nonetheless, no studies have been conducted to evaluate whether AS at these doses can be utilized clinically for the prevention and treatment of OA, and additional clinical researches are necessary to better validate the effect of AS on OA.

Our study had several limitations. First, the animal experiments in this study only evaluated the histology of articular cartilage and the expression of cartilage metabolic markers in mice, with no assessment of mouse mobility. Second, although our data suggested that AS reversed the inhibitory effects of TNF‐α on BMMSCs chondrogenic differentiation through the stabilization of YAP, the downstream mechanisms of YAP were not explored in depth. In addition, because this study was a preliminary investigative study at the cellular level and in an animal model of OA, clinical trials are still needed to further verify the preventive and therapeutic effects of AS on OA.

In conclusion, this study demonstrated that TNF‐α inhibited chondrogenic differentiation of BMMSCs by disrupting the balance of cartilage metabolism and promoting oxidative stress in BMMSCs in vitro, while AS treatment attenuated these effects. Furthermore, a detailed molecular mechanistic analysis indicated that YAP played a critical regulatory role in this process. In addition, AS treatment mitigated the progression of cartilage degeneration in a mouse DMM model. In conclusion, AS alleviated the inhibitory effects of TNF‐α on BMMSC chondrogenic differentiation by stabilizing YAP.

## AUTHOR CONTRIBUTIONS

Ziji Zhang, Puyi Sheng, and Yan Kang designed the experiments. Xudong Wang, Hongyi Liao, Yong Liu, Yunze Kang, Qingqiang Tu, and Zhiwen Li conducted the experiments. Xudong Wang and Hongyi Liao acquired the data. Xudong Wang, Hongyi Liao, Ziji Zhang, Puyi Sheng, and Yan Kang analysed the data. Xudong Wang, Hongyi Liao, Ziji Zhang, Puyi Sheng, and Yan Kang wrote the manuscript. All authors read and approved the final manuscript.

## CONFLICT OF INTEREST

All authors declare no conflict of interest.

## Supporting information


**Figure S1.** TNF‐α promotes oxidative stress of BMMSCs by downregulating YAP, while aspirin reverses these TNF‐α‐induced effects through stabilization of YAP. (A) After overexpression of YAP, expressions of YAP, NOX1, NOX2, SOD1, and SOD2 were detected by western blot. (B) After silencing the expression of YAP, the expressions of YAP, NOX1, NOX2, SOD1, and SOD2 were detected using western blot analyses. YAP, yes‐associated protein; NOX1, NADPH oxidase 1; NOX2, NADPH oxidase 2; SOD1, superoxide dismutase 1; SOD2, superoxide dismutase 2. *p < 0.05, **p < 0.01, ***p < 0.001.
**Figure S2.** The pathology images of heart, liver, spleen, and kidney in different groups mice. (A) Pathology images of heart, liver, spleen, and kidney in mice with different treatments after 8 weeks (*n* = 5 per group). (B) Pathology images of heart, liver, spleen, and kidney in mice with different treatments after 12 weeks (*n* = 5 per group). Scale bars: 250 μm (100× figures), 50 μm (400× figures).

## Data Availability

The datasets analysed during the current research are available from the corresponding author on reasonable request.
