# Supplementary material for: Aspirin reverses inflammatory suppression of chondrogenesis by stabilizing YAP
Source: Cell Prolif. 2022 Dec 10;56(4):e13380. doi: 10.1111/cpr.13380 (PMC10068956; doi:10.1111/cpr.13380)
Supplement: Supplementary file 1 — Figure S1. TNF‐α promotes oxidative stress of BMMSCs by downregulating YAP, while aspirin reverses these TNF‐α‐induced effects through stabilization of YAP. (A) After overexpression of YAP, expressions of YAP, NOX1, NOX2, SOD1, and SOD2 were detected by western blot. (B) After silencing the expression of YAP, the expressions of YAP, NOX1, NOX2, SOD1, and SOD2 were detected using western blot analyses. YAP, yes‐associated protein; NOX1, NADPH oxidase 1; NOX2, NADPH oxidase 2; SOD1, superoxide dismutase 1; SOD2, superoxide dismutase 2. *p < 0.05, **p < 0.01, ***p < 0.001. Figure S2. The pathology images of heart, liver, spleen, and kidney in different groups mice. (A) Pathology images of heart, liver, spleen, and kidney in mice with different treatments after 8 weeks (n = 5 per group). (B) Pathology images of heart, liver, spleen, and kidney in mice with different treatments after 12 weeks (n = 5 per group). Scale bars: 250 μm (100× figures), 50 μm (400× figures). [file CPR-56-e13380-s001.docx]

**Aspirin Reverses Inflammatory Suppression of Chondrogenesis by Stabilizing YAP**

**Supplementary Figures and figure legends**

**Figure S1**

**
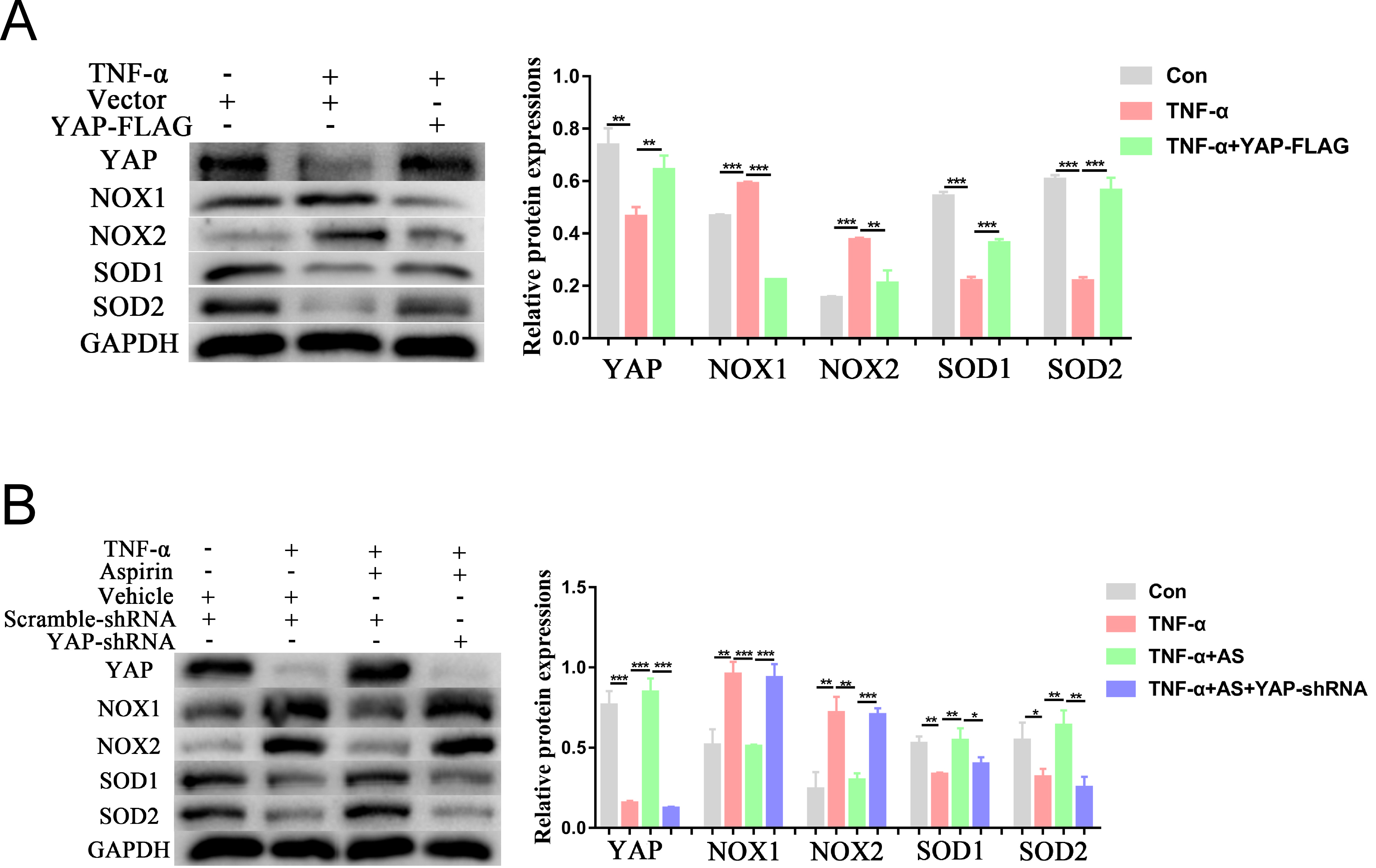
**

**Figure S1.** TNF-α promotes oxidative stress of BMMSCs by downregulating YAP, while aspirin reverses these TNF-α-induced effects through stabilization of YAP. (**A**) After overexpression of YAP, expressions of YAP, NOX1, NOX2, SOD1, and SOD2 were detected by western blot. (**B**) After silencing the expression of YAP，the expressions of YAP, NOX1, NOX2, SOD1, and SOD2 were detected using western blot analyses. YAP, Yes-associated protein; NOX1, NADPH oxidase 1; NOX2, NADPH oxidase 2; SOD1, Superoxide dismutase 1; SOD2, Superoxide dismutase 2. *P<0.05, **P<0.01, ***P<0.001.

**Figure S2**

**
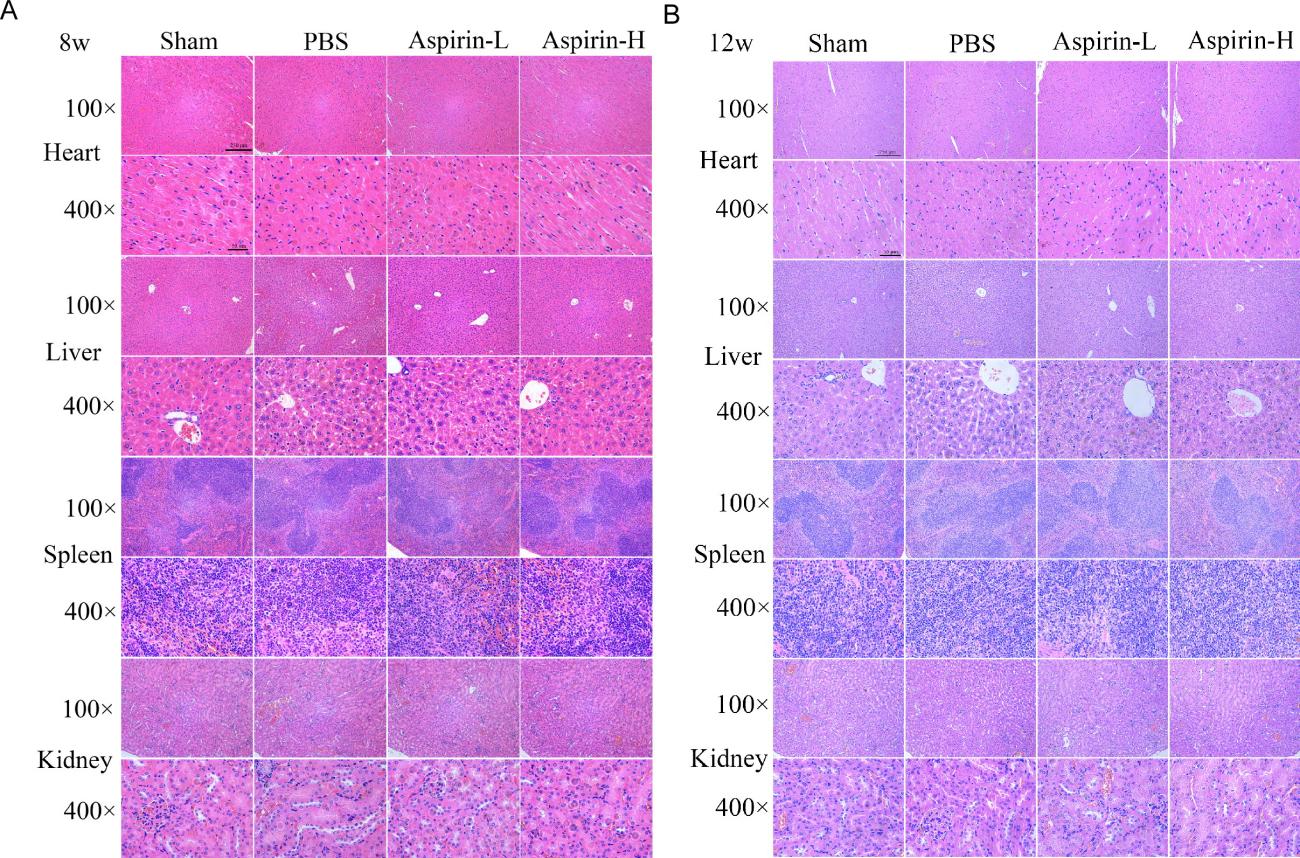
**

**Figure S2.** The pathology images of heart, liver, spleen, and kidney in different groups mice. (**A**) Pathology images of heart, liver, spleen, and kidney in mice with different treatments after 8 weeks (n=5 per group). (**B**) Pathology images of heart, liver, spleen, and kidney in mice with different treatments after 12 weeks (n=5 per group). Scale bars: 250 μm (100× Figures), 50 μm (400× Figures).
